# First Evidence of Reproductive Adaptation to “Island Effect” of a Dwarf Cretaceous Romanian Titanosaur, with Embryonic Integument *In Ovo*

**DOI:** 10.1371/journal.pone.0032051

**Published:** 2012-03-08

**Authors:** Gerald Grellet-Tinner, Vlad Codrea, Annelise Folie, Alessandra Higa, Thierry Smith

**Affiliations:** 1 The Field Museum, Chicago, Illinois, United States of America; 2 The Journey Museum, Rapid City, South Dakota, United States of America; 3 CONICET, CRILAR, Anillaco, La Rioja, Argentina; 4 Faculty of Biology and Geology, University Babeş-Bolyai, Cluj-Napoca, Romania; 5 Direction Earth and History of Life, Royal Belgian Institute of Natural Sciences, Brussels, Belgium; 6 Faculty of Math and Science Department, Oglala Lakota College, Kyle, South Dakota, United States of America; Raymond M. Alf Museum of Paleontology, United States of America

## Abstract

**Background:**

The Cretaceous vertebrate assemblages of Romania are famous for geographically endemic dwarfed dinosaur taxa. We report the first complete egg clutches of a dwarf lithostrotian titanosaur, from Toteşti, Romania, and its reproductive adaptation to the “island effect”.

**Methodology/Findings:**

The egg clutches were discovered in sequential sedimentary layers of the Maastrichtian Sânpetru Formation, Toteşti. The occurrence of 11 homogenous clutches in successive strata suggests philopatry by the same dinosaur species, which laid clutches averaging four ∼12 cm diameters eggs. The eggs and eggshells display numerous characters shared with the positively identified material from egg-bearing level 4 of the Auca Mahuevo (Patagonia, Argentina) nemegtosaurid lithostrotian nesting site. Microscopic embryonic integument with bacterial evidences was recovered in one egg. The millimeter-size embryonic integument displays micron size dermal papillae implying an early embryological stage at the time of death, likely corresponding to early organogenesis before the skeleton formation.

**Conclusions/Significance:**

The shared oological characters between the Haţeg specimens and their mainland relatives suggest a highly conservative reproductive template, while the nest decrease in egg numbers per clutch may reflect an adaptive trait to a smaller body size due to the “island effect”. The combined presence of the lithostrotian egg and its embryo in the Early Cretaceous Gobi coupled with the oological similarities between the Haţeg and Auca Mahuevo oological material evidence that several titanosaur species migrated from Gondwana through the Haţeg Island before or during the Aptian/Albian. It also suggests that this island might have had episodic land bridges with the rest of the European archipelago and Asia deep into the Cretaceous.

## Introduction

The late Cretaceous was an unusual period characterized by high eustatic sea levels, when Europe was progressively fragmented into islands of variable sizes [1]. The Haţeg Island, now part of Romania, is probably the best known, as it has been the topic of recent investigations [2]–[4]. Its vertebrate fauna displays distinct, geographically endemic dinosaur taxa (“island effect”) typified by dwarfed herbivorous titanosaurs that were remarkably primitive compared to contemporaries from other continents. Current studies recognize two dwarf lithostrotians, *Paludititan nalatzensis* [5] and *Magyarosaurus dacus* [6]–[9], although the latter may actually represent a complex of closely related nemegtosaurid species.

Recent discoveries demonstrate that lithostrotians had a worldwide distribution since the Early Cretaceous [10] and even had reached Mongolia in the Aptian/Albian [11]. However, aside from recent reports [11], [12], their paleobiology, especially their reproductive behaviors and early ontogenetic development are still a source of debate. Moreover, little is known about the reproductive behaviors of these sauropods in respect to their island adaptation.

The Haţeg Basin has been recognized as a major dinosaur nesting area during the Late Cretaceous, but the identity of these oospecies is still elusive and has been alternatively associated with hadrosaurs [13], [14] and titanosaurs [14], [15]. Adding to this confusion, the previously described dinosaur eggs from this region have been assigned to a parataxonomic and paraphyletic egg group referred as megaloolithid [14]. It was unclear, therefore, whether the now Hateg Basin nesting site was monospecific or was shared by several dinosaur species that reproduced concomitantly or sequentially.

Here we report the discovery of 11 homogenous lithostrotian egg clutches that were collected in sequentially arranged sedimentary layers within the nearly vertical outcrops of the Maastrichtian Sânpetru Formation at Toteşti [16], as well as millimeter-size embryonic integument with already formed dermal papillae inside one of these eggs. Its bacterially induced fossilization is supported by bacterial tracks and bodies in the calcium phosphate replaced integument and reinforces the importance of bacteria in soft tissue preservations [17]–[19], as previously documented for the membrana testacea of the Auca Mahuevo eggshells [20]. These Haţeg clutches represent the most complete and defined assemblage of lithostrotian eggs in Europe, as other nesting sites consist of ill-defined or isolated eggs. Furthermore, this discovery allows the first understanding of the reproductive biology of a dwarf titanosaur species. Finally, the fossils reinforce the inferred existence of Cretaceous faunal connections between South America, Europe, and Asia [21].

## Results and Discussion

### Horizon and Locality

**Figure 1:**
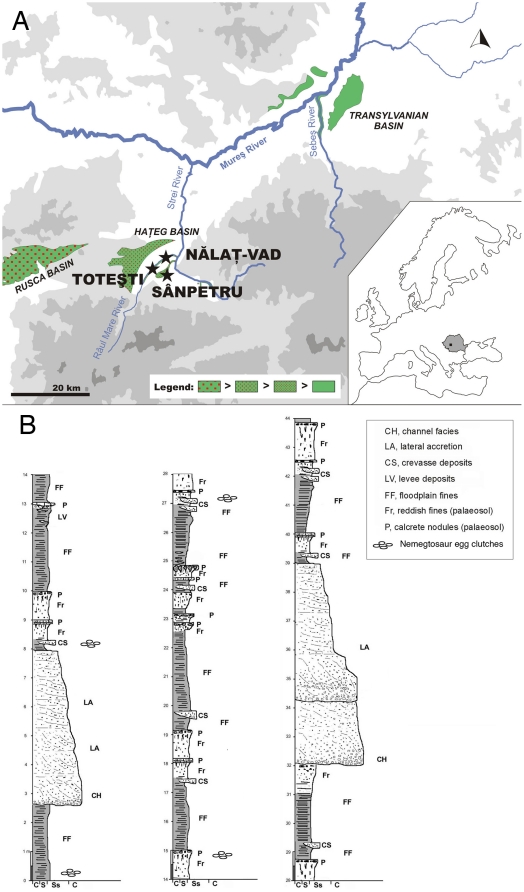
Sedimentary basins with continental Maastrichtian formations (green) in Transylvania. (*A*) Red dots show volcanic influence (ashes, cinerites, and others particular expressions within each basin), with the density of dots proportional to higher or lower volcanic influence. (*B*) Stratigraphic column of Toteşti (not inclusive of Nălaţ Vad, 3 km downstream). Note the position of the 4-egg clutches throughout the column (also noted in [16]), indicating a philopatric behavior of the dwarf island-bounded nemegtosaurids.

Forty eggs in 11 distinctive groups were collected in 2001 by a Belgo-Romanian team [16] from Toteşti-baraj, where the Maastrichtian-aged Sânpetru Formation [22], [23] is exposed in the Râul Mare River bed (Fig *1A*). Although these specimens were previously regarded as nests [16], we defined them here as clutches because no true nesting structures were observed in the encasing sediments. In contrast to the poorly sorted grains with large clasts (indicating high energy) of the sedimentary layer, where eggs and associated dinosaur embryos were previously reported [13] at Tuştea, the exposures at Toteşti consist of very thin-grain sedimentary layers (thin siltstone-mudstone), and thus imply autochthonic assemblages within a low energy depositional environment.

Our field observations corroborate the presence of relatively thin mudstone strata with a 75–80 N dip and a (N40-50 E) strike roughly parallel to the riverbanks [16], [24] with small, interspersed isolated siltstone lenses. As such, an aerial view of the exposure corresponds to a vertical section, and the clutches have been vertically rotated from their original position. The 4 egg-clutches were recovered in sequential strata [16], which facilitated their diagnosis as homogenous and separate assemblages (Fig. *1B*). However, it became more difficult to differentiate one clutch from another in one instance where 2-3 assemblages were positioned in the same sedimentary layer and at the exact same geographical coordinates. As such, the 4-egg clutches, which were originally ovideposited on the same horizontal sedimentary surface, became superposed because of the dipping of the strata. Regardless of this atypical occurence, the presence of these clutches in several and consecutive strata strongly suggests a philopatric behavior by the same dinosaur species (Fig. *1B*).

More eggs and eggshell fragments of different appearances and thicknesses were collected by the authors in 2010 between the original 2001 site and Nălaţ-Vad, another fossiliferous locality of similar age and geology 3 km downstream from Toteşti [24], [25]. Yet, it is important to specify that these fossils were isolated, not in defined clutches, and consisted only of isolated eggshell fragments of various size; thus it is not the intent of this study to identify these other specimens. This material was sparse and dispersed throughout the Râul Mare River sedimentary layers, which are rarely completely exposed and often transected by numerous faults [16], [25], making accurate geological and age correlations difficult [16].

### Egg taxonomic identity

**Figure 2:**
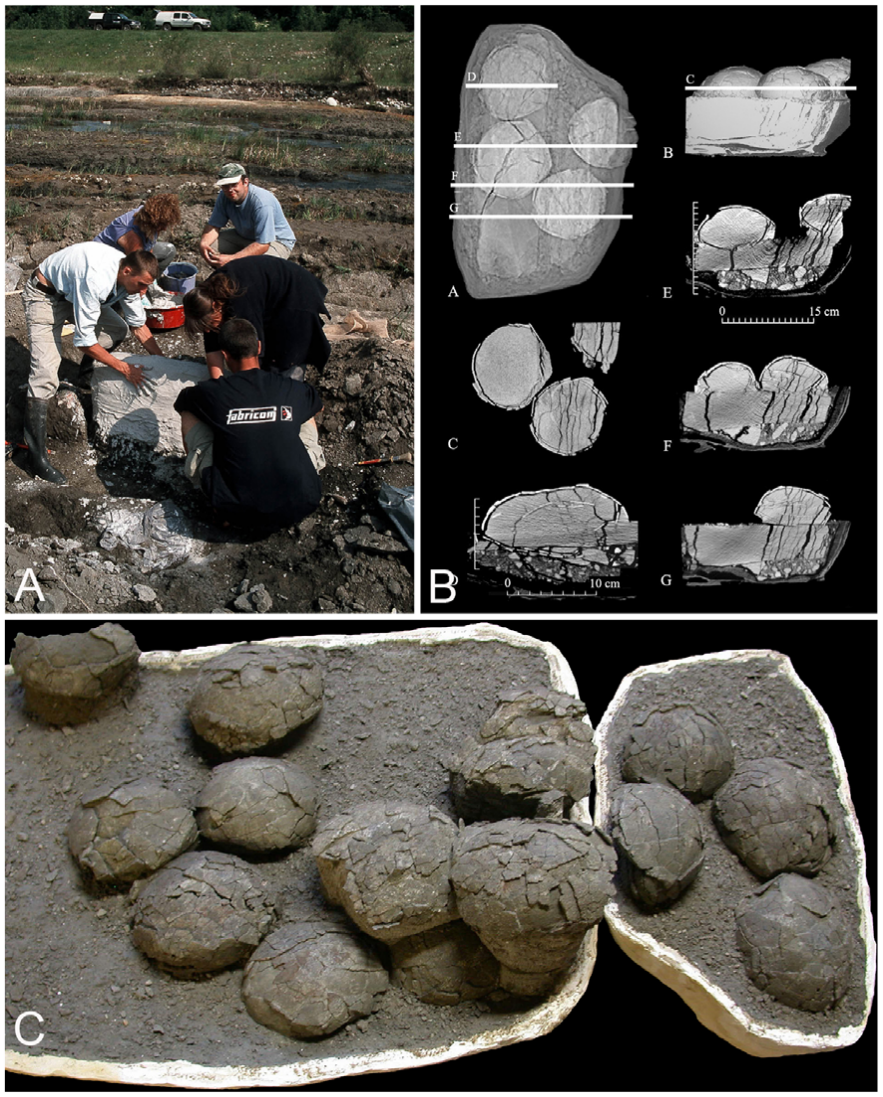
Field work and egg clutches assemblages. (*A*) Field work in the Râul Mare river bed at Toteşti, showing that the jacketed clutch was vertically rotated, with a dip of 75–80 N. (*B*) The 11 clutches consist of sets of 4–5 lithostrotian eggs. Due to minor compaction, the measurements of the nearly spherical eggs of clutch TO O–01 were obtained digitally and range between 11 and 13 cm in diameter. These measurements match exactly those from the positively identified Auca Mahuevo titanosaur eggs. (*C*) An assemblage of 3 clutches (TO O–01; IRSNB Cast-Vert 32) now on exhibit at the University of Cluj and the Royal Belgian Institute of Natural Sciences. The eight partially prepared remaining clutches are housed at the University of Cluj.

Due to minor compaction (Fig. *2B, and C*), the nearly spherical eggs are moderately fractured and range between 11 and 13 cm in diameter (Fig. *2B*). These digital measurements were obtained from the CT scan of clutches TO O–01 (Fig. *2A, and C*) now on exhibit at the University of Cluj and the Royal Belgian Institute of Natural Sciences. They are consistent with caliper measurements of partially prepared egg clutches of the same assemblages that are presently housed at the University of Cluj. These dimensions substantially differ from previous reports of 14–16 cm diameter eggs [16] and slightly exceed those of the Asian lithostrotian egg with its embryo [10], but match exactly those from the positively identified Auca Mahuevo titanosaur eggs [20], [26]. Eggshell microcharacterization rests on scanning electron microscopy (SEM), energy dispersion spectrometry (EDS), and transmitted light microscopy (TLM). More than 50 eggshell samples were selected from clutch TO O–01, including several specimens from the other clutches of the same assemblage (TO O–03, TO O–04) as well as eggshell fragments from single eggs (from these clutches) to test any possible eggshell variations. In addition, the newly collected orphan eggshells in 2010, as above-mentioned, were also included in our observations. Eggshell thickness is constant throughout the clutch samples and equals 1.7 to 1.8 mm, but greatly differs from the previous report of 2.14–2.82 mm [*16*] and isolated oological material collected in 2010.

Eggs and their eggshells are biomineralized systems, similar to skeletal systems, thus display specific phylogenetic characters [27], [28]. Hence, their inclusion in trace fossil parataxonomic classifications is inaccurate and at best misleading. In addition, phylogenetic analyses based on oological characters have proved to mirror those resting on skeletal features [29]. Thorough oological description without *a priori* inclusion in parataxonomic classifications would supply enough phylogenetic characters to obtain evolutionary hypotheses for this biomineralized system. However, the majority of past descriptions suffers from a parataxonomic insertion and/or lack such detailed and complete descriptive sections, thus limiting possible oological analyses. Moreover, to date, only two assemblages with sauropod embryos allow sauropod oological phylogenetic analyses to be anchored to their parent lineages: Auca Mahuevo, Argentina [26] and Ulan Tsaav, Mongolia [11].

**Figure 3:**
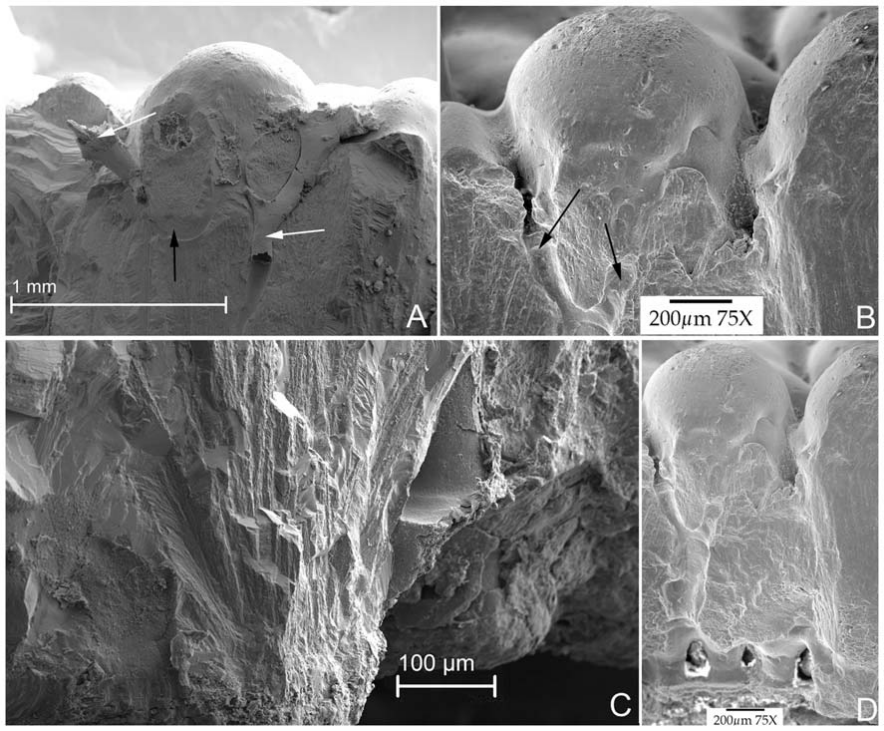
Identification and comparison of the Toteşti and Auca Mahuevo specimens. (*A* and *C*) SEM of Toteşti specimen. (*B* and *D*) SEM of Auca Mahuevo specimen from egg bearing level 4. (*A*) Toteşti eggshell from the prepared clutch TO O–01. (*B*) Eggshell from Auca Mahuevo, egg-bearing level 4. Note the complete similarities (pore size, shape, position, node size and concentration, in addition to a similar 1.7 to 1.8 mm shell thickness) between the materials from these two countries. However, the Toteşti material is invaded by palygorskyte inclusions. Black arrows point to palyygorskyte included in pore canals, white arrows to pore canals. Note the Y-shaped canals in both the Patagonian and Romanian specimens. (*C*) SEM of base of Toteşti TO O–01 specimen. *C* displays the same acicular radiating crystals that are characteristic of the Patagonian material. Moreover, the presence of a horizontal pore canal network parallel to the membrana testacea that was first described in the Patagonian eggshells also occurs in Toteşti material, as seen in *C*. The triangular shape of the canal apertures is the same and positioned at the same topological level as observed in the Patagonian specimens in (*D*).

**Figure 4:**
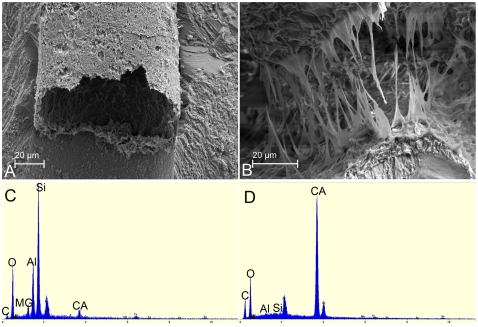
Palygorskyte inclusion in Toteşti eggshells. (*A–B*) SEM of palygorskyte inclusions in TO O–01 pore canals. (*C–D*) EDS of palygorskyte and eggshell respectively. Note that in *A*, the pristine palygorskyte forms tubular inclusions in the eggshell pore canals, a condition never reported before. (*B*) Magnification of *A*. Note the crystallographic habit of this sample that is different from that from high evaporitic conditions. This particular habit indicates either that it derives from smectite or directly originates from hydrothermal precipitations, thus suggesting indirectly the presence of hydrothermal or tectonic activities. The latter is corroborated by the presence of lenticular travertine rich in strontium and barium and coeval regional intracratonic volcanic activities. (*C*) Palygorskyte EDS shows a high Si peak, substantial Al and O concentrations with minor Mg presence, all typical for clay minerals. (*D*) Conversely, the eggshell EDS displays a strong Ca peak followed by a notable presence of O and minor amount of C, indicative of calcium carbonate. The Au peak is present in both EDS because the samples were gold coated.

**Figure 5:**
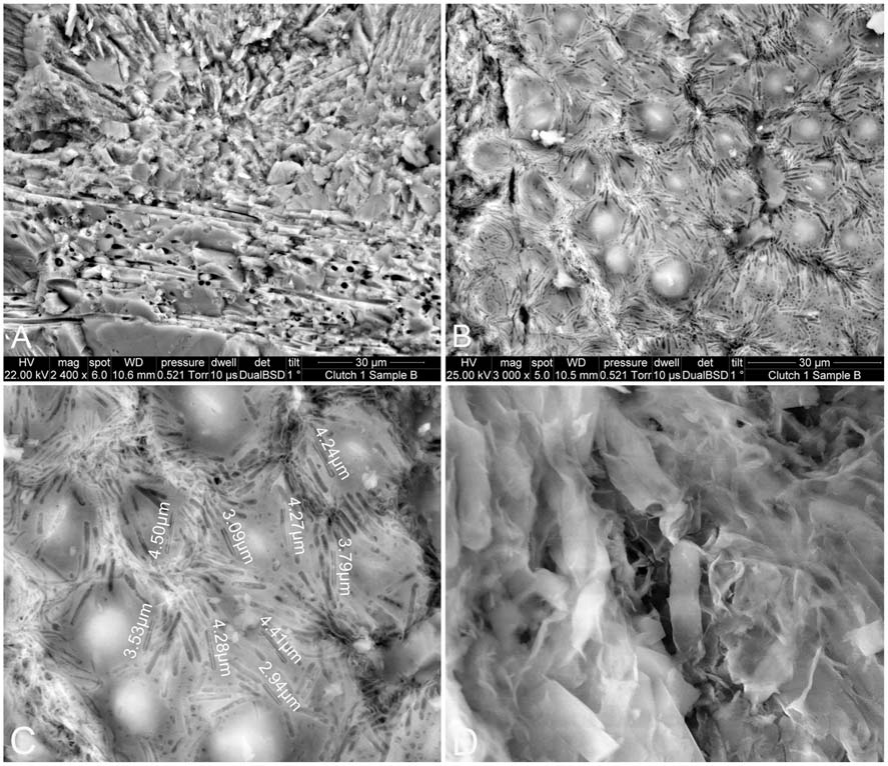
(*A–C*) Egg and embryonic soft tissues. SEM of TO O–03 specimens. (*A*) Note that the membrana testacea fossilized parallel strands in the TO O–03 egg that preserved the embryonic skin. EDS observations indicate only presence of calcium carbonate. (*B*) A fossilized section of integument with bacterial remains and tracks and dermal papillae in the shape of non-overlapping minuscule semi-elongated to round domes, which diameters vary between 8 and 12 µm. (*C*) Magnification of *B*. Note the proliferation of bacterial tracks on and in between the domes. The fossilization of this integument is clearly bacterially induced. Such soft tissue fossilization could result in mobilization of phosphate where its concentration is sufficient to inhibit or prohibit the precipitation of calcium carbonates and may even be promoted in closed systems, as in closed eggs. (*D*) Presence of ∼4 *µm* long fossilized bacteria confirms the shape and length of the tracks observed throughout the integument and further demonstrates the importance of bacteria in soft tissue preservations.

In view of these restrictions, our comparative observations rest on oological material that has been completely described without *a priori* biases and the two instances where embryos were recovered *in ovo*. Interestingly and unexpectedly, the oological characters of these Romanian clutches (Fig. *S1*) are totally congruent with those of eggs from Auca Mahuevo egg-bearing level 4 (Fig. *3*), which was previously unreported but illustrated by Grellet-Tinner et al. ([26]: figure *4*, specimen MCF-PVPH 444). Synapomorphies encompass egg size and shape and external and internal eggshell morphological and microstructural characters (Fig. *3*). Specifically, characters include identical egg shape and size, eggshell thickness, and radial sections of the shell display a single structural layer consisting of acicular calcitic crystals radiating from nucleation centers (Fig. *3C*) located similarly above the membrana testacea (MT). In between each unit, a conspicuous series of pore canals above the MT between each eggshell unit (Fig. *3C*) forms a network parallel to the MT regularly connected to multiple vertical pore canals [26], [30]. The atypical Y-shaped vertical pore canals (Fig. 3*A*) open in between the ubiquitous nodular surficial ornamentation in between which funnel shaped pore apertures are located. Nodes average 0.6–7 mm in diameter, as previously reported by Codrea et al. [16]. Each separate branch of these Y-shaped canals joins into the wider lower section of the Y-shaped canals. Several vertical canals are filled by a hollow material never reported before in such conditions (Fig. 3*A*). EDS combined with observations at higher SEM magnifications indicates the presence of palygorskyte (Fig. *4*), a clay derived from evaporitic systems [31], from smectite [31]–[34], or hydrothermal precipitation [35]. In addition, the MT consists of a fibrous mat that is exceptionally well-preserved in several specimens (Fig. *5A*), and its thickness (0.2 mm) is nearly identical to that of the specimens from Auca Mahuevo egg-bearing level 4 (0.2–0.25 mm).

In sum, the overwhelming oological evidence places the eleven Toteşti clutches in the same clade as those from Auca Mahuevo egg-bearing level 4 [26]. In addition to oological evidence, the latest review of the Auca Mahuevo embryos *in ovo* ([36]: 426) identifies several cranial characters that are congruent with the nemegtosaurids *Quaesitosaurus* [37], *Nemegtosaurus* [38], and *Rapetosaurus* [39]. This taxonomic assignment fits with the known Haţeg titanosaur faunal assemblage, consisting of the lithostrotian *Paludititan nalatzensis* and most commonly the nemegtosaurid *Magyarosaurus dacus*. As such, the total phylogenetic evidence (skeletal and oological) strongly supports identification of the 11 studied egg clutches as Nemegtosauridae.

### Paleobiology and paleoenvironment

This first described set of well-preserved and complete titanosaur egg clutches from the Maastrichtian of Romania is critical to developing a better insight into the adaptive nesting strategies of dwarf lithostrotians (inclusive of nemegtosaurids) and their paleobiology to the “island effect”. The oological data, as a whole, strongly support similarity with the Auca Mahuevo 4^th^ and youngest egg-bearing level specimens [26]. The number of well-delimited clutches and their occurrence in sequential strata argue for nesting site fidelity, perhaps even philopatric behavior. Yet, this discovery has other several profound implications. The first but not the least is that Auca Mahuevo, similarly to the Toteşti site, might not have been a monospecific nesting site, but may have serially hosted several titanosaur species throughout its four distinct egg-bearing levels. This inference rests on the difference between the oological material from the Auca Mahuevo egg-bearing levels 3 [26] and 4. Second, regardless of the egg-bearing level, the number of eggs per clutch at Auca Mahuevo exceeds 15 eggs per assemblage in previous reports [40]. The drastic difference in number of eggs per clutch between the continental species and its island congener suggests that dwarf titanosaurs may have adapted their reproductive biology to the “island effect” by drastically decreasing the number of eggs per clutch rather than reducing the egg size or/and changing the eggshell structural features. This interesting adaptation is congruent with an analysis of clutch size in relation to body size of modern crocodylians performed in the context of this study (Fig. *S2*). Results show a significant correlation between clutch size reduction with a decrease in body size, which mirrors the pattern observed between the continental (Auca Mahuevo) and island (Haţeg) titanosaurs. Conversely, such clutch size reduction cannot be related to whether the eggs were buried or ovideposited on the substrate surface, because the phylogenetic characters of these specimens related to pore conductance express a functional morphological adaptation to extremely high moisture level that could only be achieved in buried nests, as demonstrated by Deeming [30], [41].

The oological disparities between previous reports [14], [16], [25] and these observations are substantial, yet could be easily explained. Previous reports mention larger eggs with thicker eggshell in Haţeg [14], [16], [25]. Our 2010 fieldwork in Transylvania confirms the presence of isolated and broken specimens throughout the Toteşti and Nălaţ-Vad exposures, with eggshells as thick as 2.8 mm (Fig. *S3*). Although the studied eggshells and the thicker shelled eggs share a few characters that are superficially similar, detailed microcharacterizations (Fig. *S3*) reveal significant differences in their eggshell unit shapes, pore network arrangements and concentrations, which are noticeably more developed in the thicker eggshells of larger isolated eggs. Such notable oological disparities typify the presence of several egg-laying dinosaur species throughout the time represented by the successive Râul Mare River exposures. Whether these various species ovideposited their eggs concomitantly or sequentially remains uncertain and complicated by the faulting system that in several instances cuts and displaces the nearly vertical exposures of the Râul Mare River [16], [25]. Regardless, the undisputable presence of at least two large egg species (Fig. *S3*) in the river strata between Toteşti and Nălaţ-Vad provides evidence that this site had favorable ecological settings that attracted several dinosaur species [13]–[15] to ovideposit their eggs in a limited region of the Cretaceous Haţeg Island.

Reproduction in oviparous vertebrates is more constrained by environmental factors than in their viviparous counterparts. Judicious nesting site selection is therefore critical, as parents cannot compensate post-hatching for a poor choice of nesting environment [12], [30], [42]–[44]. Such selectivity, even to the extent of precise location of nests within preferential nesting sites [45], [46], affects hatching success and developmental rates. In addition, nesting-site philopatry, a behavior wherein offspring return as adults to their own site of birth to nest [47], [48], exacerbates the sensitivity of suitable nesting site choice to environmental changes. The importance of nesting microenvironments is furthermore illustrated by opportunistic nesting in geothermal settings [12], [49]–[54]. This represents an interesting adaptive case where species avoid thermally heterogeneous nesting environments and exploit the geothermal conditions to maintain ideal temperatures and moisture levels in egg clutches, thus demonstrating the importance of finding optimum environments in respect to eggs and their eggshells.

The discovery of pristine palygorskyte inclusions in eggshell pore canals (Figs. *3A* *and* *4*), which has never been reported previously, has profound ecological implications. Palygorskyte, in principle, could be formed in high evaporitic conditions, and displays a unique crystal habit when originating in this environment. However, palygorskyte can also derive from smectite [31], [34] or directly originate from hydrothermal precipitations [35]. In these latter two circumstances, the clay exhibits crystal habits similar to those observed in the eggshell inclusions, thus suggesting indirectly the presence of geothermal/hydrothermal, tectonic, or volcanic activities, which coincidently were concomitant with the Sânpetru Formation. Independently, limestone lenses rich in eggshells and microvertebrate remains in Nălaţ-Vad [25], which were constrained to a single stratum of the Sânpetru Formation, have been discovered. Geochemical analyses reveal the presence of excessive amount strontium and barium concentrations and other rare earth elements (Fig. *S4*) similar to those of well-diagnosed travertine deposits [55]. These results strongly suggest the occurrence of an active geothermal system in Haţeg Basin during the Maastrichtian, which is further and independently supported by coeveal intracratonic volcanic activity in the southwestern Carpathians and Apuseni Mountains [56]–[58].

Vertebrate eggs require species-specific moisture and heat to ensure successful hatching, which could be achieved through various strategies. Paleogeothermal settings in Haţeg Basin thus could have provided such stable and optimal local nesting conditions, explaining the occurrence of eggs from several dinosaur species in an otherwise restricted geographically island environment. Such reproductive behaviors on specific insular volcanic fields are still reported in modern amniotes. For instance, the megapodes are known to exploit geothermal resources in the South Pacific islands [46]. In addition, dinosaur reproduction linked to geothermal field has already been well documented [12], [30]. Moreover, both the Totesti studied eggs and the thicker-shelled isolated specimens exhibit extensive and intricate pore system that facilitate water vapor conductance and gas exchanges, in turn reflecting elevated nesting moisture levels [41]. As previously argued [30], the Y-shaped pore canals that abut in a secondary pore canal system located at and above the MT level surrounds the embryo [26], [30] and creates a biomechanical system that enhances gas exchanges with the highly vascularized corioalantoid membrane, similar to the specialized trachea and lungs of the respiratory system [30], [59], [60]. Such a pore network system would favor greater gas diffusion between the embryo and its elevated nesting moisture environment without comprising the egg's mechanical integrity.

### Soft tissue preservation

One specimen from clutch TO O–03, still partially in its original 2001 plaster jacket, displays remarkable tissue preservation, with the parallel strands of the membrana testacea (MT) fossilized and perforated by minute transversal fibers (Fig. *4A*). Detail is exquisite, as expressed by the delicate micron size fossilization of the protein strands that composed the MT (Fig. *5A*), mirroring the bacterially-induced structures in the Auca Mahuevo eggs [20]. Further inspection of TO O-03 revealed the unexpected fossilization of another soft tissue riddled with bacterial tracks (Figs. 5*B and C*) that are uniform in width but vary from 2.94 to 4.50 µm in length. In addition, a few bacterial bodies were observed on the same surface (Fig. *5D*), thus justifying a bacterially-induced fossilization mode. The fossilized tissue consists of non-overlapping, minuscule semi-elongated to round domes, with diameters varying between 8 and 12 µm (Figs. *5B and C*).

Early organogenesis in amniotes is characterized by the formation of the dermis, followed by skeletal initiation. In reptiles, a scale contains a single prominent melanotic spot, over which the epidermis is raised in the form of a dome-shaped papule over a locally thickened area of dermis [61]. Specifically, the development of scales in squamate reptiles begins also with epidermal papillae, in the form of undulations of the epidermal surface producing symmetric dermo-epidermal elevations [62]. In *Alligator mississippiensis*, dermal papillae start with larger bundles that consist of 3 nm thick electron-pale keratin microfibrils that increase in size with ontogenetic development [63]. Aside from a thickened appearance, osteoderm precursors do not differ histologically or histochemically from the surrounding matrix [64].

The size and shape of the domes in TO O-03 is congruent with dermal papillae in modern crocodilians at ontogenetic stage 17 [65]. Therefore, our present knowledge of embryology suggests that the dome-shaped features observed in TO O–03 are dermal papillae, preserved through bacterially-induced fossilization. This exquisite fossilization is perfectly congruent with that of the MT. Moreover, the micron size of these dome–shaped tubercles implies that the embryo died at a very early embryological stage, before skeletal ossification. The fossilization of millimeter-size embryonic skin riddled with bacterial tracks (Fig. *5B–D*) inside TO O–03 mirrors previous descriptions of the nemegtosaurid embryonic skins from Auca Mahuevo [66], but at an unprecedented earlier ontogenetic stage. Thus, TO O–03 represents a miniature version of the 800 µm domes originally described in the Auca Mahuevo eggs [67]. Furthermore, the occurrence of these domes in a Haţeg lithostrotian taxon is congruent with the presence of osteoderms of the armoured nemegtosaurid *Magyarosaurus dacus* [7].

Energy dispersion spectrometry (EDS) indicates a high concentration of calcium phosphate in this tissue (Fig. *S5*), in contrast to the calcium carbonate of the rest of the eggshell. Bacterially-induced fossilization of this integument, here supported by bacterial bodies and tracks in the calcium phosphate, is entirely congruent with similar occurrences documented in the fossil record [17]–[20] and clay mineralogy [68]. Yet, the presence of apatite raises an interesting question in respect to the role of bacteria in soft tissue fossilization. Bacterially-induced soft tissue fossilization could result in mobilization of phosphate, where its concentration is sufficient to inhibit or prohibit the precipitation of calcium carbonates, and which may even be promoted in closed systems [18]. Eggs are, indeed, perfect closed systems with an ample reserve of organic material in their yolk for instance, where clay minerals, which seal them from extrinsic factors, could induce and favor such biochemical reactions. Micron size organic replacement and phosphatization by bacteria is common in Lagerstätten [18] and was previously described for the muscle fibril fossilization in a few Las Hojas fossils [17], [19]. In addition, preserved fish bones in this Lagerstätte display the same bacterial tracks as the Haţeg embryonic integument ([19], Fig. *3E*).

### Paleogeography

The new egg clutches help clarify the biogeographic distribution of Late Cretaceous European titanosaurs. It has long been hypothesized that the Haţeg Island supported endemic faunas that arose through *in situ* diversification of Early Cretaceous lineages that were stranded in Europe as sea levels rose [69]–[71]. However, this view has been recently challenged by the recent discovery of a bizarre dwarf theropod, *Balaur bondoc* [3]. We offer here an alternative hypothesis.

The occurrence of a lithostrotian titanosaur in the Aptian-Albian of Mongolia [11] suggests that this clade had already achieved a global distribution at that time, and by the same token a Gondwana-Laurasia connection occurred between the Hauterivian ([10]) and Aptian. However, the only known Aptian nemegtosaurid is from the Quirico Formation of the Brazilian San Franciscana Basin [10]. The fossil record, combined with phylogenetic analyses, supports the hypothesis that Nemegtosauridae originated in the Barremian [10] but achieved a widespread distribution during the Late Cretaceous, including the two known Asian nemegtosaurids, *Quaesitosaurus orientalis* [37] and *Nemegtosaurus mongoliensis* [72]. The presence of *Paludititan nalatzensis* [5] and *Magyarosaurus dacus* [6]–[9] in the Haţeg Island is congruent with successive early radiations of lithostrotian titanosaurs from Gondwana.

Contrary to previous notions, then, Haţeg Basin only would have been intermittently isolated from the rest of the European archipelago and Asia during successive cycles of regression and transgression during the middle Lower Cretaceous. Yet this scenario, with Barremian nemegtosaurid migration from South America, is incongruent with the timing of the complete separation of Gondwana from Laurasia, as discussed by Wilson and Upchurch [73], but would support later ephemeral Gondwana-Laurasia connections [21], [70], [74] until the mid Lower Cretaceous [75], [76]. Nemegtosaurids would have saltated through the European archipelago from micro continents to islands during episodic regressive events between the Aptian and Campanian, to reach Asia. Whether this titanosaur clade became stranded during sporadic faunal interchange between the European archipelago and Asia (which would have persisted long into the Cretaceous, as supported by recent discoveries of dinosaur and mammal fauna [3], [77]–[81]), or during the original Gondwana-Laurasia migrations, is still open for discussion.

## Materials and Methods

Toteşti and Nălaţ-Vad eggs are catalogued with the appellation of TO O and NV O, respectively. As such, the egg assemblages on display in Cluj and in Brussels are TO O–01 and IRSNB Cast-Vert 32 respectively. This assemblage consists of 3 distinct clutches that were not recognized at the time of discovery, due to the Toteşti geology. Eggshell specimens from various clutches have the same names as the clutch followed by an added alphabetic letter. Microcharacterizations of the eggshell specimens were conducted at the ACMM Center of the University of Sydney, Australia, and the Royal Belgian Institute of Natural Sciences, Brussels, Belgium. Examinations of the specimens were performed with and without coating. Geochemical analyses were conducted at the SARM of the CRPG, Nancy, France.

## Supporting Information

Figure S1 **Taxonomic characters of the new Toteşti eggs.** Comparison of the eggs from the 11 clutches with 7 other localities. Note that the greatest amount of similar characters is presently shared by the new eggs and the Auca Mahuevo specimens.
(XLS)

Figure S2 **The relationship between the size of 23 crocodilian species and their respective egg clutch size.** The relationship between the size of 23 crocodilian species (data from [82]) and their respective egg clutch size was tested with SPSS linear regression. The size of the 23 crocodilian species varied between 1.7 to 7 m (4.1±SE 0.39) and the egg clutch average size between 12.5 to 55 eggs (32.59±SE 2.93). All of the data points fall within the 95% prediction limits, except for one species, and the regression is significant with a strong correlation (*R^2^* = 0.62, *P*<0.001). These results indicate that larger crocodiles lay more eggs per clutch, with a ratio of 10 eggs for 1 m increase in body length on the average, thus indicating a positive and strong correlation between body length and clutch size.
(DOCX)

Figure S3 **Comparison of several isolated oological remains in the Râul Mare River beds.** (*A–B*) SEMs of thick eggshell found in the Râul Mare River beds between Toteşti and Nălaţ-Vad. They are mostly isolated and very fragmentary. Although the eggshell structure shares a few similarities with those from the 11 clutches, thicknesses of these isolated specimens could reach 2.8 mm. (*C*) TLM observations at the same scale of eggshell from clutch TO O–01 (top) and an isolated specimen from the Râul Mare River bed (below). Note the greater thickness of the bottom specimen and a higher concentration of pore canals. The top specimen is thinner but complete, as attested by the presence of a capping layer of secondary calcitic deposit on its outer surface.
(TIF)

Figure S4 **Geochemical analysis of travertine specimens.** The travertine lenses do not cross cut entirely the Sanpetru Formation. Results indicate substantial strontium and barium concentrations that confirm a geothermal origin, which is expected because coeveal intracratonic volcanic activities were occurring in the southwestern Carpathians and Apuseni Mountains during the Maastrichtian.
(XLS)

Figure S5 **Comparison between the elemental composition of the embryonic integument and the eggshell.** Microanalysis supports the elevated concentration of Ca, P, and O in TO O–03, which contrasts with the rest of the eggshell, solely composed of Ca and O. As indicated, O, P, and Ca represent 39.92, 16.22, and 41.76 elemental weight percent of the specimen. The presence of calcium phosphate is attributed to the bacterial mobilization of phosphate, where and when its concentration is promoted even in closed systems such as unhatched eggs.
(TIF)
