# Supplementary material for: First Evidence of Reproductive Adaptation to “Island Effect” of a Dwarf Cretaceous Romanian Titanosaur, with Embryonic Integument In Ovo
Source: PLoS One. 2012 Mar 8;7(3):e32051. doi: 10.1371/journal.pone.0032051 (PMC3297589; doi:10.1371/journal.pone.0032051)
Supplement: Figure S2 — The relationship between the size of 23 crocodilian species and their respective egg clutch size. The relationship between the size of 23 crocodilian species (data from [82]) and their respective egg clutch size was tested with SPSS linear regression. The size of the 23 crocodilian species varied between 1.7 to 7 m (4.1±SE 0.39) and the egg clutch average size between 12.5 to 55 eggs (32.59±SE 2.93). All of the data points fall within the 95% prediction limits, except for one species, and the regression is significant with a strong correlation (R2 = 0.62, P<0.001). These results indicate that larger crocodiles lay more eggs per clutch, with a ratio of 10 eggs for 1 m increase in body length on the average, thus indicating a positive and strong correlation between body length and clutch size. (DOCX) [file pone.0032051.s002.docx]

| **Genus** | **Species** | **species size (m)** | **number of eggs per clutch** | | |
| --- | --- | --- | --- | --- | --- |
|  |  |  | **Average** | **minimum** | **maximum** |
| *Alligator* | *A. mississippiensis* | 5 | 40 | 30 | 50 |
|  | *A. sinensis* | 2 | 25 | 10 | 40 |
| *Caiman* | *C. c. crocodilus* | 2.8 | 22.5 | 15 | 30 |
|  | *C. latirostris* | 2.5 | 45 | 20 | 70 |
|  | *C. yacare* | 2.5 | 29.5 | 21 | 38 |
| *Melanosuchus* | *M. niger* | 5 | 47.5 | 30 | 65 |
| *Paleosuchus* | *P. palpebrosus* | 1.7 | 12.5 | 10 | 15 |
|  | *P. trigonatus* | 2.3 | 15 | 10 | 20 |
| *Crocodylus* | *C. acutus* | 7 | 45 | 30 | 60 |
|  | *C. intermedius* | 6.8 | 55 | 40 | 70 |
|  | *C. johnsoni* | 3 | 13 |  |  |
|  | *C. mindorensis* | 2.5 | 15 | 10 | 20 |
|  | *C. moreletii* | 2.5 | 30 | 20 | 40 |
|  | *C. niloticus* | 7 | 52.5 | 25 | 80 |
|  | *C. novaeguineae* | 3.4 | 33.5 | 22 | 45 |
|  | *C. palustris* | 4 | 27.5 | 25 | 30 |
|  | *C. porosus* | 7 | 50 | 40 | 60 |
|  | *C. rhombifer* | 4.9 | 35 | 30 | 40 |
|  | *C. siamensis* | 4 | 35 | 20 | 50 |
| *Mesistops* | *M. cataphractus* | 4 | 16 |  |  |
| *Osteolaemus* | *O. tetraspis* | 2 | 15 | 10 | 20 |
| *Gavialis* | *G. gangeticus* | 7 | 40 | 30 | 50 |
| *Tomistoma* | *T. shlegelii* | 5 | 40 | 20 | 60 |
