# Supplementary figures and images for: First Evidence of Reproductive Adaptation to “Island Effect” of a Dwarf Cretaceous Romanian Titanosaur, with Embryonic Integument In Ovo
Source: PLoS One. 2012 Mar 8;7(3):e32051. doi: 10.1371/journal.pone.0032051 (PMC3297589; doi:10.1371/journal.pone.0032051)

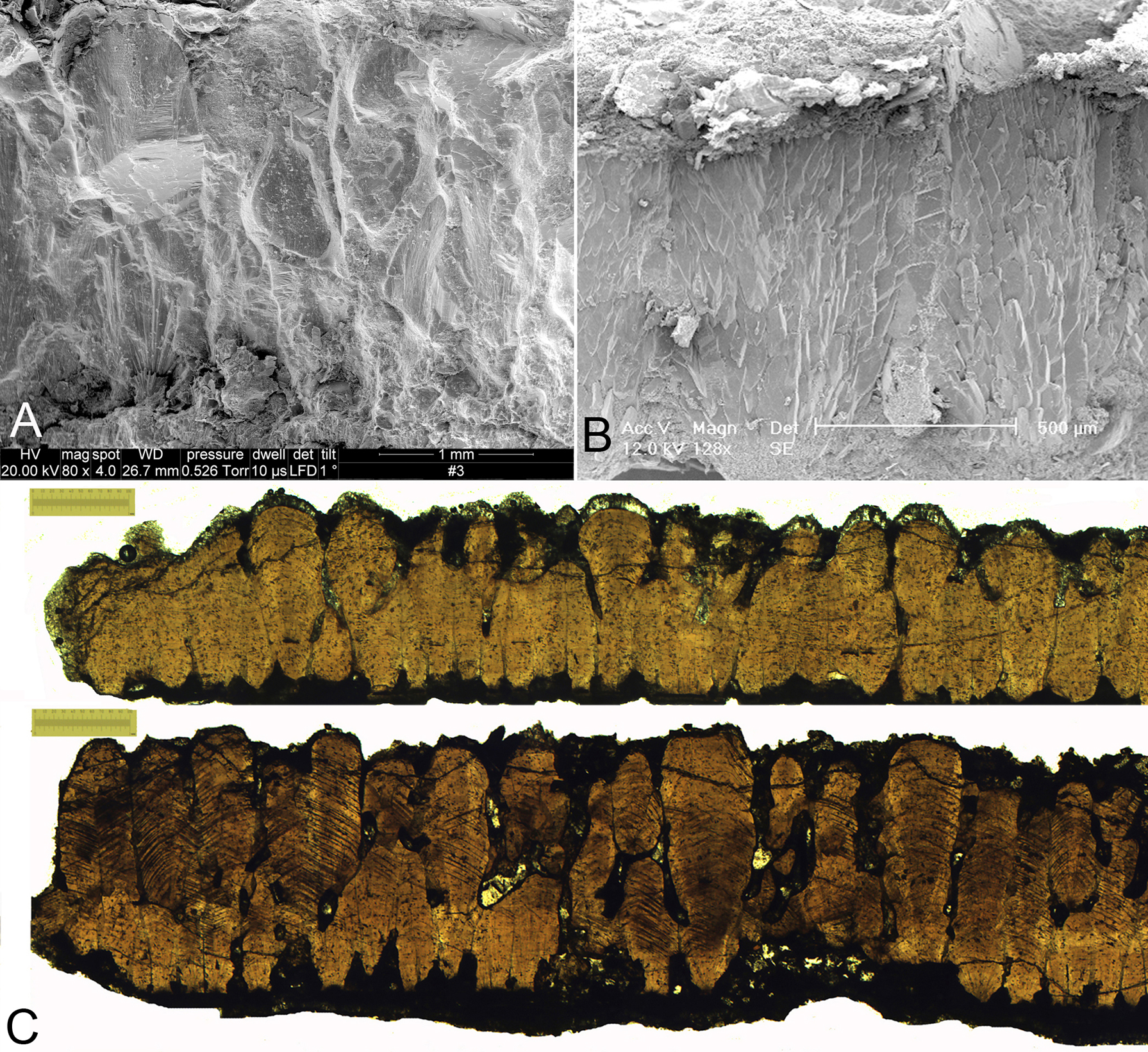

Supplement: Figure S3 — Comparison of several isolated oological remains in the Râul Mare River beds. (A–B) SEMs of thick eggshell found in the Râul Mare River beds between Toteşti and Nălaţ-Vad. They are mostly isolated and very fragmentary. Although the eggshell structure shares a few similarities with those from the 11 clutches, thicknesses of these isolated specimens could reach 2.8 mm. (C) TLM observations at the same scale of eggshell from clutch TO O–01 (top) and an isolated specimen from the Râul Mare River bed (below). Note the greater thickness of the bottom specimen and a higher concentration of pore canals. The top specimen is thinner but complete, as attested by the presence of a capping layer of secondary calcitic deposit on its outer surface. (TIF) [file pone.0032051.s003.tif]

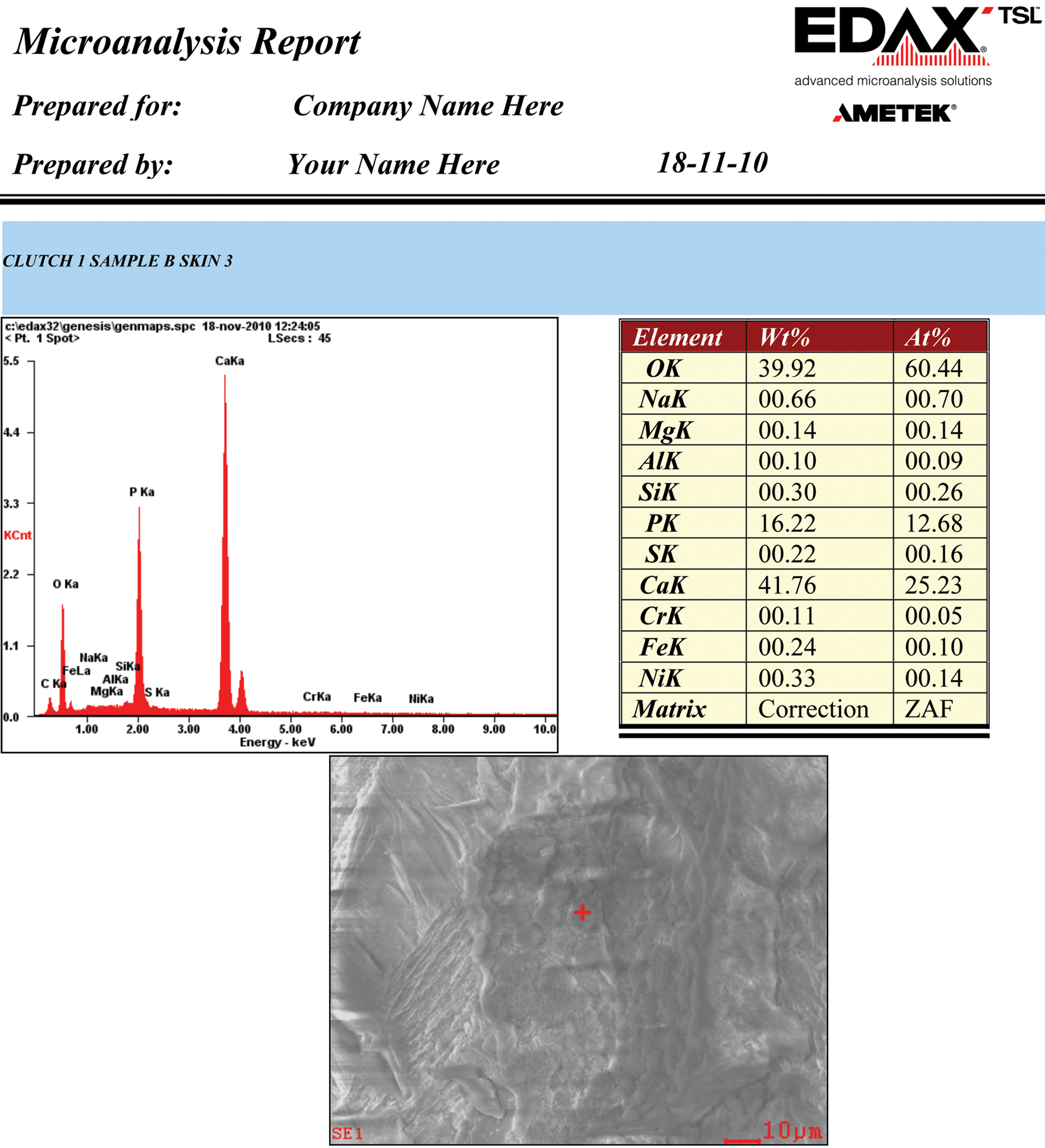

Supplement: Figure S5 — Comparison between the elemental composition of the embryonic integument and the eggshell. Microanalysis supports the elevated concentration of Ca, P, and O in TO O–03, which contrasts with the rest of the eggshell, solely composed of Ca and O. As indicated, O, P, and Ca represent 39.92, 16.22, and 41.76 elemental weight percent of the specimen. The presence of calcium phosphate is attributed to the bacterial mobilization of phosphate, where and when its concentration is promoted even in closed systems such as unhatched eggs. (TIF) [file pone.0032051.s005.tif]
